# Validation of an endoscopic flavectomy training model

**DOI:** 10.1590/0100-6991e-20202901

**Published:** 2021-04-24

**Authors:** ÁLYNSON LAROCCA KULCHESKI, EDMAR STIEVEN-FILHO, CAROLLINE POPOVICZ NUNES, PAUL ANDRÉ ALAIN MILCENT, LEONARDO DAU, XAVIER SOLER I-GRAELLS

**Affiliations:** 1 - Universidade Federal do Paraná (UFPR), Departamento de Cirurgia - Ortopedia e Traumatologia - Curitiba - PR - Brasil

**Keywords:** Medical Education, Simulation Training, Endoscopy, Spine, Ligamentum Flavum, Educação Médica, Treinamento Por Simulação, Endoscopia, Coluna Vertebral, Ligamento Amarelo

## Abstract

**Objective:**

to validate a lumbar spine endoscopic flavectomy simulator using the construct method and to assess the acceptability of the simulator in medical education.

**Methods:**

thirty medical students and ten video-assisted surgery experienced orthopedists performed an endoscopic flavectomy procedure in the simulator. Time, look-downs, lost instruments, respect for the stipulated edge of the ligamentum flavum, regularity of the incision, GOALS checklist (Global Operative Assessment of Laparoscopic Skills), and responses to the Likert Scale adapted for this study were analyzed.

**Results:**

all variables differed between groups. Procedure time was shorter in the physician group (p < 0.001). Look-downs and instrument losses were seven times greater among students than physicians. Half of the students respected the designated incision limits, compared to 80% of the physicians. In the student group, about 30% of the incisions were regular, compared to 100% in the physician group (p < 0.001). The physicians performed better in all GOALS checklist domains. All the physicians and more than 96% of the students considered the activity enjoyable, and approximately 90% believed that the model was realistic and could contribute to medical education.

**Conclusions:**

the simulator could differentiate the groups’ experience level, indicating construct validity, and both groups reported high acceptability.

## RESUMO

**Objetivo:**

validar um simulador de flavectomia endoscópica da coluna lombar por meio do método de constructo e, analisar a aceitação do simulador no ensino médico.

**Métodos:**

trinta estudantes de medicina e dez ortopedistas com experiência em videocirurgia realizaram um procedimento de flavectomia endoscópica no simulador. Foram analisados tempo, look-downs, perdas de instrumentos, respeito ao limite estipulado no ligamento amarelo, contorno regular do corte, checklist GOALS (Global Operative Assessment of Laparaoscopic Skills) e respostas à Escala de Likert adaptada para este estudo.

**Resultados:**

todas as variáveis diferiram entre os grupos. O tempo do procedimento foi menor no grupo dos médicos (p < 0,001). Look-downs e perdas de instrumentos foram sete vezes superiores entre os alunos do que entre os médicos. Metade dos alunos respeitou os limites de incisão designados, em comparação a 80% dos médicos. No grupo dos alunos, cerca de 30% das incisões foram regulares, em comparação a 100% no grupo dos médicos (p < 0,001). Os médicos tiveram melhor desempenho em todos os domínios da checklist GOALS. Todos os médicos e 96% dos alunos consideraram a atividade prazerosa, e cerca de 90% consideraram que o modelo era realista e poderia contribuir para o ensino médico.

**Conclusões:**

o simulador foi capaz de diferenciar o nível de experiência dos grupos, indicando a validade do construto, e ambos os grupos relataram alta aceitação.

## INTRODUCTION

Spinal endoscopy emerged in the 1990s as a less invasive method for the surgical treatment of herniated lumbar discs. This technique has functional advantages over traditional methods^1^ ^-^ ^3^. However, it requires a greater learning curve than the open technique^4^.

Surgical simulators could shorten the learning curve and improve the safety of surgeons in a new technique^5^. However, the progress of surgical performance must be assessed, which can be based on specific surgical acts as well as on specific checklists such as GOALS (Global Operative Assessment of Laparoscopic Skills), which assesses five skills of video-assisted surgical fitness^6^ ^-^ ^8^. Moreover, for surgical training, simulators must be validated, with the main methods being: face validity, content validity, construct validity, concurrent validity, and transfer validity. The construct validity method, commonly used to evaluate a simulator, is based on a previously defined variable. It is expected that experienced surgeons have greater surgical skills than those who do not perform surgery, and the better performance of surgeons in a simulator should be evident^9^ ^-^ ^12^. 

The use of objective criteria, such as procedure time and the number of instrument losses or look-downs, gives credibility to the analysis. The more often the operator looks down, the greater the difficulty in understanding the three-dimensional environment of a video-assisted surgery and the less skilled the operator is^13^ ^,^ ^14^.

Questionnaires such as those involving Likert scales are also used to corroborate the research results^15^.

Using simulators of spinal endoscopy is an interesting option, since endoscopic flavectomy is not widely used and its practice requires repetition^1^ ^-^ ^3^. However, the prototypes currently available on the market are expensive, limiting access to training.

To fill the niche between expensive commercial simulators and low-cost solutions, this study aimed to validate a reproducible simulator of endoscopic lumbar spine flavectomy using the construct method, the GOALS checklist, and Likert scale assessments. The simulator’s acceptability in medical education was also assessed.

## METHODS

This cross-sectional experimental study was approved by the research ethics committee of a university hospital (reference 1,994,655).

The sample consisted of a group of orthopedists and a control group of senior medical students, who were selected through simple random sampling using a random number generator (UX APPS Random Number®, version 2.1.8.2018). The exclusion criteria were refusal to grant consent and previous contact with the simulator. 

**Figure 1:**
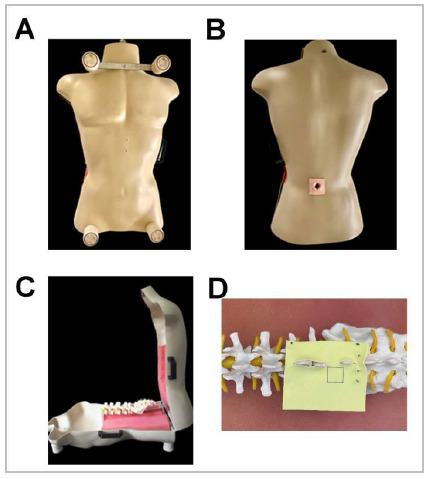
A) Frontal view. B) Dorsal view. C) Open simulator - lateral view. D) Yellow ligament placed in the vertebral model.

A previously developed spinal endoscopy simulator was used to assess the selected individuals^16^ (Figure 1).

The yellow ligament was simulated using an 8 x 11 cm sheet of yellow ethylene-vinyl acetate with a 6.25 cm^2^ square drawn on it (Figure 1). 

**Figure 2:**
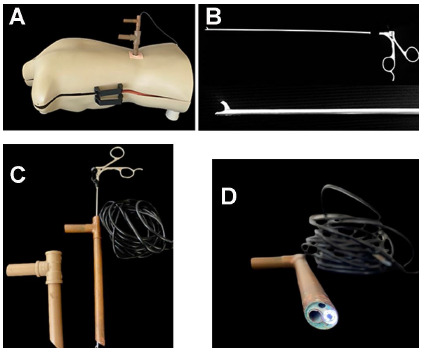
The endoscope used in the simulator with the following instruments: A) Endoscope inserted in the simulator. B) Scissors used in the flavectomy procedure. C) Tube system, endoscope and scissors. D) Endoscope light source.

An SXT-5.0M video camera (KKMOON, Shenzhen, China) coupled to a computer was used to simulate the endoscope, with the images projected on a monitor. The probe-type camera had its own light source and USB port (Figure 2). 

Real endoscopic surgical scissors were used for the flavectomy procedure (Figure 2).

The simulator’s cost was US$90.00 (BRL$465,00)^16^.

All participants were instructed about the model’s function and the procedure to be performed through a 5-minute video. The video demonstrated the handling technique for the endoscopic forceps, anatomical concepts of the lumbar spine and the endoscopic opening procedure for the yellow ligament.

The participants were positioned in front of the simulator (placed on an 80 cm high table) with a frontal view of the image projection screen and instructed to begin the simulated flavectomy.

The participant inserted the instruments into the simulator through the classic dorsal paramedian portal at the L5-S1 level. The participant was asked to find his/her position in the space, identify the surrounding structures, the mark on the yellow ligament, and perform the flavectomy up to designated limit in the model (maximum 6.25 cm^2^), opening the ligament in a rectilinear manner in the center of the marked square, so that the nerve root of L5-S1 and the herniated disc (represented in red) could be visualized.

All procedures were supervised, and the participants were told to interrupt the activity if they considered the result satisfactory or when the 10-minute time limit was reached. All procedures were performed at the Orthopedic Skills Laboratory of our institution.

**Figure 3:**
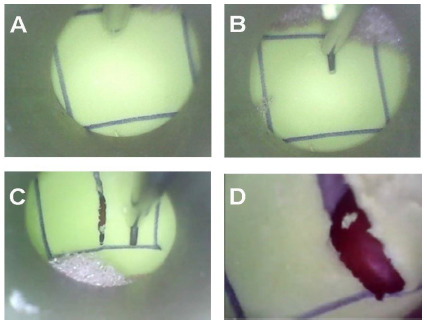
Endoscopic image of the flavectomy. A) Identifying the limits of the flavectomy. B) Beginning the flavectomy with the endoscopic scissors. C) Correctly centralized and finalized flavectomy. D) Identifying the herniated disc (red).

The endoscopic images from the flavectomy were transmitted to a personal computer via USB cable and recorded with NCH Debut 5.14.c video capture software (NCH Software, Inc., Greenwood Village, CO, USA) (Figure 3). 

External video of the participants was recorded with an iPhone 7 (iOS 12.4.1) on a tripod positioned one meter away from the participant (Supplementary Material 1).

Supplementary Material 1
External view of the procedure.

Individual photographs of the yellow ligament used in each procedure were taken before and after the flavectomy with an iPhone 7 (iOS 12.4.1) on a tripod set vertically 20 cm over the ligaments. The ligaments were not identified and were compared for integrity, regularity, and respect for the stipulated limits (Supplementary Material 2).

Supplementary Material 2
A) Regular flavectomy B) Irregular flavectomy.

The videos and images were analyzed blindly. The assessed variables were total procedure time, number of look-downs, and number of instrument losses, respect for the established limit for the yellow ligament (exceeding this limit was considered inappropriate; this item was rated as “yes” or “no”), and an appropriate, regular incision shape (i.e., straight and centered without deviations and having delicate edges). 

The endoscopic and external videos were analyzed according to the GOALS method^8^. At the end of the test, the evaluator issued a score up to a maximum of 25 points.

At the end of the procedure, the participants answered a two-part questionnaire (Supplementary Materials 3, 4, 5), containing the following elements: demographic data (name, sex, age, and video-assisted surgery experience) and a Likert scale questionnaire on the participant’s impressions about the simulator and its applicability in medical education. Each group was asked five questions, which were responded on a 5-point scale from “totally disagree” to “totally agree”. The following questions were asked:

Group I (physicians)

1. Was simulator training a motivating/enjoyable activity?
2. Does the simulator facilitate recognition of the anatomical structures encountered in a real surgery?
3. Would the simulator be useful for training surgeons who are new to the technique?
4. Can the simulator replace cadaver training?
5. Do you consider yourself capable of performing a real endoscopic flavectomy?

Group II (students)

1. Was simulator training a motivating/enjoyable activity?
2. Do you feel that the simulator stimulated learning in your coursework?
3. Did the instructions help you execute the task?
4. Would you like to undergo simulator training in other areas of orthopedics?
5. Do you feel that the format and design of the simulator are realistic?

Supplementary Material 3
Demographic data.

Supplementary Material 4
Likert scale (medical students).

Supplementary Material 5
Likert scale (orthopedists).

Qualitative variables were represented as absolute and relative frequencies. Quantitative variables and scores were represented by median and interquartile range (first quartile; third quartile). The chi-square and Mann-Whitney tests were used to compare qualitative and quantitative variables, respectively. 

Sample size calculation was based on the analysis of the power of statistical tests^17^.The sample size was calculated based on previous studies with the same methodology that used the standard deviation of the variable ‘time’ to detect differences in the magnitude of 1.05 standard deviation between the groups, with 80% power and 95% confidence level^18^. 

The significance level was set at 5%. All analyses were performed in Microsoft Excel® (2013) and R® 3.4.4 (R Foundation for Statistical Computing Vienna, Austria).

## RESULTS

The sample consisted of 30 students (53% female, 47% male) and ten orthopedists (100% male), who accounted for the total number of physicians with sufficient experience to perform the procedure safely. The 30 students were randomly selected from the population of 88 students enrolled in the last year of the medical curriculum in our institution. Mean age was 23 years for students and 44 years for physicians. Physicians’ mean experience in video-assisted surgery was 13 years. Five orthopedists had experience in knee arthroscopy, three in shoulder arthroscopy, one in knee and shoulder arthroscopy, and one in knee, ankle, and shoulder arthroscopy.

The quickest procedure, less than 2 minutes, was performed by the physician with 9 years of experience in knee and shoulder arthroscopy. Two students used the full 10-minute allowance to complete the task and another five students required more than eight minutes to do so, while none of the doctors required the maximum time. The difference between the mean total time of the students (4 min) and doctors (2 min) was significant (p <0.001).

The Mann-Whitney test was used to compare the groups of variables monitored throughout the procedure. The significance level was set at 5% (Table 1).

**Table 1:** Analytical statistics of objective visual parameters.

Variable	Students	Physicians	p-valor
Number of participants	30	10	
Total time (minutes)	4 (3.5; 6)	2 (2; 2.7)	<0.001
Look-downs (quantity)	7 (5; 9)	1 (0; 1)	<0.001
Instrument losses (quantity)	7 (5; 9)	1 (0; 2)	<0.001
Respect of the ligament limits	16 (53.3%)	8 (80%)	0.26
Appropriate contour at the limits	10 (33.3%)	10 (100%)	0.001
Surgical experience (years)	-	13 (9; 19)	-

The physician group had lower values for all parameters. All physicians obtained an appropriate contour at the edges, while only one-third of the students did so.

The GOALS score was lower for the student group in every domain, as well as in the consolidated total. We found a statistically significant difference between the groups for all assessed competences (Table 2). 

**Table 2:** Comparison of GOALS parameters between students and physicians.

GOALS	Students	Physicians	p-value
Depth perception	1 (1; 3)	5 (5; 5)	<0.001
Bimanual dexterity	2 (1; 3)	5 (5; 5)	<0.001
Efficiency	1 (1; 3)	5 (5; 5)	<0.001
Tissue handling	1 (1; 3)	5 (5; 5)	<0.001
Autonomy	1 (1; 3)	5 (5; 5)	<0.001
Total	8 (5; 13)	25 (25; 25)	<0.001

The distribution (as percentages) of the participants’ responses to the Likert Scale is shown in Table 3.

**Table 3:** Likert scale responses.

	Students				
Question	Totally disagree	Partially disagree	Neither disagree nor agree	Partially agree	Totally agree
1 - Is simulator training a motivating activity?	0	0	3.3%	33.3%	63.4%
2 - Does the simulator stimulate your learning in the discipline?	0	0	16.7%	30%	53.3%
3-Did the instructions help you complete the task?	0	3.3%	0	36.7%	60%
4 - Would you like to train with simulators in other areas of orthopedics?	6.7%	0	6.7%	30%	56.6%
5 - Do the format and design look like the real thing?	0	3.3%	16.7%	56.7%	23.3%
	Physicians				
Question	Totally disagree	Partially disagree	Neither disagree nor agree	Partially agree	Totally agree
1 - Is simulator training a motivating activity?	0	0	0	40%	60%
2 - Can you recognize the anatomical structures involved in a real surgery?	0	0	0	70%	30%
3 - Is it useful for training surgeons who are new to the technique?	0	0	0	20%	80%
4 - Does the simulator replace cadaver training?	20%	30%	30%	10%	10%
5 - Do you consider yourself capable of performing a real endoscopic flavectomy?	30%	10%	30%	30%	0

## DISCUSSION

Few studies have investigated simulated endoscopic procedures of the spine^19^. Most of these studies have not used an objective validation method for their analysis^20^ ^,^ ^21^.

Among studies on low-cost simulators for video-assisted surgery training, Cunha et al. developed a simulator involving virtual reality glasses and analyzed the video-assisted procedure performed in a transparent box that allowed real-time observation by the evaluator, but without transmitting or capturing the procedure image^22^. The simulator in the present study also used accessible materials, but we opted for an opaque plastic mannequin so that the participant could only access internal structures through the endoscopic camera. 

Considering the factors evaluated during the use of the simulator, tactile feedback refers to the ability to reproduce the sensation created by applying force on natural tissue with a specific pattern of resistance. Although this characteristic is more evident in simulations in cadavers, it can also occur in synthetic simulators. Some commercial synthetic models for laparoscopy have hardened tissue consistency, requiring sudden movements to perform dissection maneuvers^23^. The present study confirmed the difficulty of faithfully reproducing the anatomical structures of soft tissues. The plastic mannequin, despite having a similar shape, has a different density and malleability than human tissue. This issue was partially resolved by filling the mannequin with foam, which reproduced the musculature and satisfactorily guided the procedure. The endoscopic scissors did not require maintenance during the study. It was difficult to reproduce the procedure faithfully with copper and PVC pipes. However, a similar but larger instrument was obtained. Nevertheless, the participant’s grip, the camera image, and the shape and distance of the endoscope in relation to the mannequin were very close to reality. The limitations of the external aspects of the model were overcome by the use of a good quality endoscopic camera used in conjunction with cell phones and computers, which allowed adequate visibility of the internal structures.

Among studies that compared the performance of different groups in simulators, Mattei et al.^13^ used a synthetic pediatric lumbar spine model and compared groups with high and low experience, with a methodology similar to that used in the present study. Both studies showed that the use of objective parameters and validated checklists lends credibility to the simulator validation process.

When a prototype is validated, the parameters must be simple, and the data must be easy to interpret and collect. Previously validated parameters were adapted to endoscopic flavectomy^17^ ^,^ ^24^ ^,^ ^25^. 

Among the evaluated parameters, task performance time is considered the most uniform metric for comparing surgical skills^26^ ^,^ ^27^. In the present study, time differentiated the groups; the physicians performed the procedure in half the time it took the students. In addition, two students used the entire time allowance to complete the task, while none of the physicians did so. This finding is similar to that of other simulator validation studies in the literature, which demonstrated an estimated 60% longer procedure time in the inexperienced group^18^ ^,^ ^27^ ^,^ ^28^.

In addition to manipulation of the simulator itself, it is also important to evaluate task performance. Milcent et al. assessed the amount of area removed from the menisci using software that provided precise measurements^18^. In the present study, the regularity of the incision and compliance with the stipulated ligament limit were assessed through visual verification and blindly to minimize assessment bias. Regarding compliance with the ligament limit, it was considered to have been exceeded or not, without quantifying the overshoot. The simplicity of this method distinguished between the two groups without requiring a specialist or specific software.

The participants were also evaluated using the GOALS method^8^. In all analyzed domains, there were significant differences between the physicians and the students, which complemented the construct validity. The GOALS checklist can be applied in future spinal endoscopy studies to assess performance progress, either in simulated training or the real surgical environment.

A number of studies have shown the advantages of using simulators in medical education^23^. In the present study, the Likert questionnaire results demonstrated that the simulator was well evaluated by about 94% of the participants, regarding its motivational quality, their interest in undergoing further simulated training in other surgical areas, and the realism of the prototype. More than 90% of the participants felt that the simulator could improve medical education due to its ability to stimulate student involvement, and the physicians recognized its potential for training young surgeons.

At the end of the procedure, only 30% of the physicians considered themselves able to perform a flavectomy using a real endoscopy, which demonstrates that isolated practice does not necessarily lead to proficiency. A study on simulator training with a decompression technique for the posterior cervical spine found that repetitive practice led to improved skill assessment scores for all participants^7^. It is believed that this simulator can demonstrate the acquired proficiency in future prospective studies assessing skill progression.

In the present study, we asked about whether the simulator replaces cadaver training, and 80% of physicians disagreed that such a replacement was appropriate. It is well known that simulation in cadavers provides greater realism to the procedure, in addition to other more effective features, such as tactile feedback^13^ ^,^ ^29^. However, the ethical issues and increasing difficulties involved in cadaver training must be considered.

The simulator proved to be effective due to its low cost, easy reproduction, and portability. Its validation involved parameters that had been previously used in the literature and were well adapted for this study. The procedure was easily understood by the participants and the main hypothesis of the study was proven, i.e. that the model could differentiate experienced and inexperience groups of participants, providing construct validity to the simulator. 

The present study has certain limitations. The number of participating physicians was limited by the number of available experienced professionals. However, the analysis of the power of statistical tests determined that the sample of ten orthopedists and 30 students was sufficient to detect differences in the magnitude of 1.05 standard deviation, considered acceptable^17^. An intermediate experience group (e.g., orthopedics residents) was not tested, although it would be interesting for future studies with the model. Performance progression was not an objective of this study and was not measured. This study did not determine whether skills were acquired through use of the simulator, or whether laboratory-acquired benefits translated into the real surgical environment, which should motivate future studies on its applicability in surgical training. 

At the time of publication, no similar studies could be found in the literature that involved such a validation methodology for endoscopic flavectomy.

## CONCLUSIONS

The spinal endoscopic flavectomy simulator could differentiate two distinct experience groups (students and orthopedists), demonstrating the construct validity of the simulator. Procedure time for physicians was half that for students, who looked down and lost the instrument on the screen seven times more often than physicians. The simulator was accepted by 94% of the participants, and 90% felt it should have a role in medical education.
