# Supplementary figures and images for: Validation of an endoscopic flavectomy training model
Source: Rev Col Bras Cir. 2021 Apr 24;48:e202027910. doi: 10.1590/0100-6991e-20202901 (PMC10683459; doi:10.1590/0100-6991e-20202901)

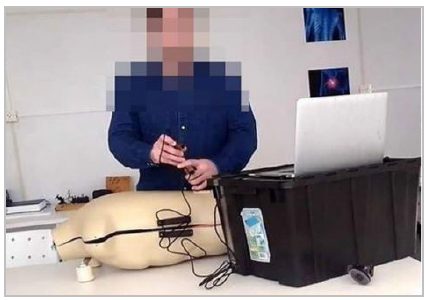

Supplement: Supplementary Material 1 [file rcbc-48-e202027910-g004.tif]

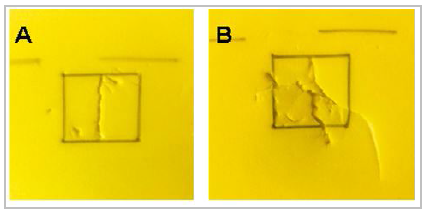

Supplement: Supplementary Material 2 [file rcbc-48-e202027910-g005.tif]

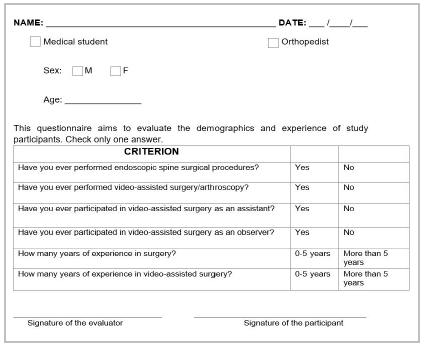

Supplement: Supplementary Material 3 [file rcbc-48-e202027910-g006.tif]

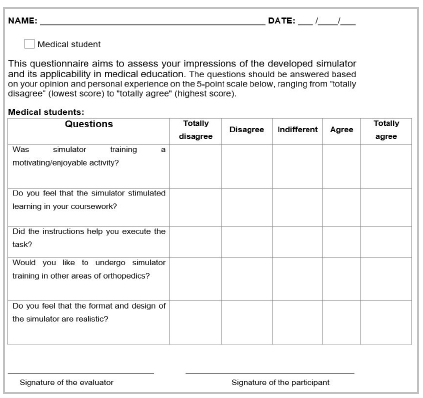

Supplement: Supplementary Material 4 [file rcbc-48-e202027910-g007.tif]

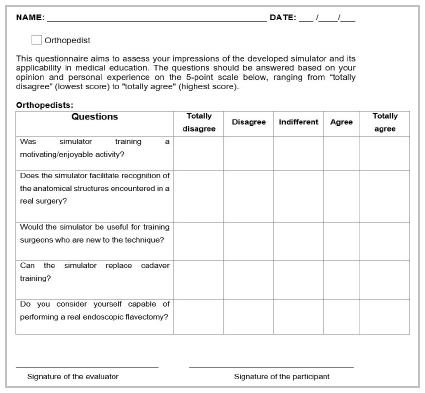

Supplement: Supplementary Material 5 [file rcbc-48-e202027910-g008.tif]
